# Pirimicarb: 2-dimethylamino-5,6-dimethylpyrimidin-4-yl dimethyl­carbamate

**DOI:** 10.1107/S160053681002684X

**Published:** 2010-07-14

**Authors:** Hojin Yang, Tae Ho Kim, Yong Woon Shin, Ki-Min Park, Jineun Kim

**Affiliations:** a Department of Chemistry and Research Institute of Natural Sciences, Gyeongsang National University, Jinju 660-701, Republic of Korea; b Test & Analytical Laboratory, Korea Food & Drug Administration, 123-7 Yongdang-dong, Busan 608-829, Republic of Korea

## Abstract

In the title compound, C_11_H_18_N_4_O_2_ (systematic name: 2-dimethyl­amino-5,6-dimethyl­pyrimidin-4-yl *N*,*N*-dimethyl­carb­amate), the pyrimidine ring and dimethyl­amino group are almost in the same plane, making a dihedral angle of 1.6 (1)°. The dihedral angle between the mean plane of the pyrimidine ring and that of the dimethyl­carbamate group is 83.42 (5)°. In the crystal structure, inter­molecular C—H⋯O hydrogen bonds contribute to the stabilization of the packing.

## Related literature

For the toxicity and insecticidal properties of the title compound, see: Pirisi *et al.* (1996 ▶). For related structures, see: Dalpozzo *et al.* (2001 ▶); Madre *et al.* (2008 ▶).
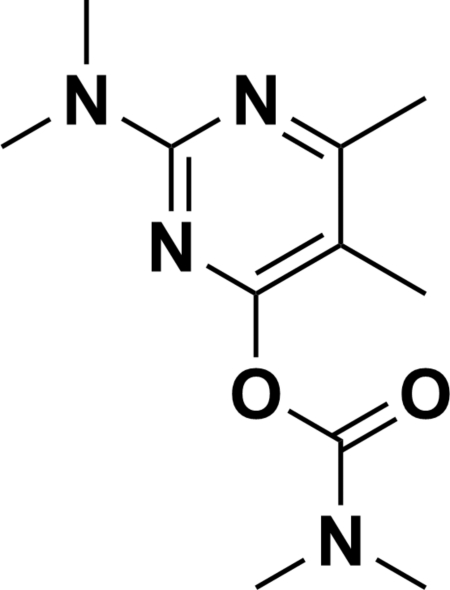


## Experimental

### 

#### Crystal data

C_11_H_18_N_4_O_2_
*M* *_r_* = 238.29
Monoclinic, 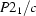

*a* = 13.5607 (7) Å
*b* = 7.7868 (4) Å
*c* = 13.1323 (7) Å
β = 114.907 (3)°
*V* = 1257.72 (11) Å^3^
*Z* = 4
Mo *K*α radiation
μ = 0.09 mm^−1^
*T* = 173 K
0.29 × 0.25 × 0.11 mm

#### Data collection

Bruker APEXII CCD diffractometer
Absorption correction: multi-scan (*SADABS*; Sheldrick, 1996 ▶) *T*_min_ = 0.975, *T*_max_ = 0.990
11979 measured reflections
3093 independent reflections
2390 reflections with *I* > 2σ(*I*)
*R*_int_ = 0.031

#### Refinement

*R*[*F* ^2^ > 2σ(*F* ^2^)] = 0.048
*wR*(*F* ^2^) = 0.146
*S* = 1.05
3093 reflections
160 parameters
H-atom parameters constrained
Δρ_max_ = 0.27 e Å^−3^
Δρ_min_ = −0.26 e Å^−3^

### 

Data collection: *APEX2* (Bruker, 2006 ▶); cell refinement: *SAINT* (Bruker, 2006 ▶); data reduction: *SAINT*; program(s) used to solve structure: *SHELXTL* (Sheldrick, 2008 ▶); program(s) used to refine structure: *SHELXTL*; molecular graphics: *SHELXTL* and *DIAMOND* (Brandenburg, 1998 ▶); software used to prepare material for publication: *SHELXTL*.

## Supplementary Material

Crystal structure: contains datablocks global, I. DOI: 10.1107/S160053681002684X/sj5033sup1.cif

Structure factors: contains datablocks I. DOI: 10.1107/S160053681002684X/sj5033Isup2.hkl

Additional supplementary materials:  crystallographic information; 3D view; checkCIF report

## Figures and Tables

**Table 1:** Hydrogen-bond geometry (Å, °)

*D*—H⋯*A*	*D*—H	H⋯*A*	*D*⋯*A*	*D*—H⋯*A*
C5—H5*C*⋯O2^i^	0.98	2.60	3.549 (2)	163
C10—H10*C*⋯O2^ii^	0.98	2.51	3.431 (2)	157

Symmetry codes: (i) 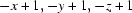; (ii) 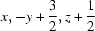.

**Fig. 1.**
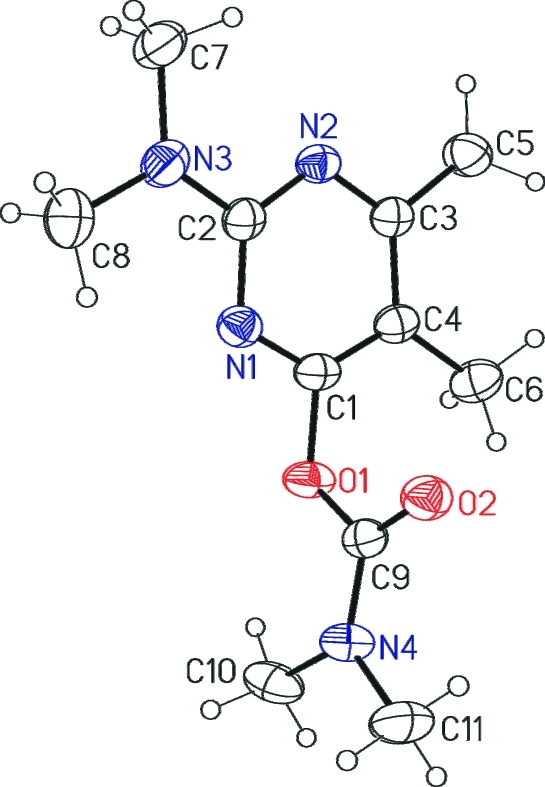
The molecular structure of the title compound, showing displacement ellipsoids drawn at the 50% probability level.

**Fig. 2.**
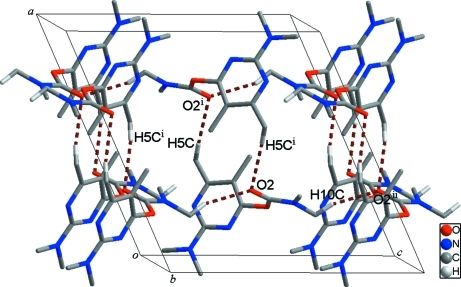
Crystal packing of the title compound with hydrogen bonds shown as dashed lines. H atoms not involved in intermolecular interactions have been omitted for clarity.

## supplementary crystallographic information

### Comment

Pirimicarb (systematic name: 2-dimethylamino-5,6-dimethylpyrimidin-4-yl dimethylcarbamate), is a well known insecticide used to control aphids on vegetable, cereal and orchard crops by inhibiting acetylcholinesterase activity (Pirisi *et al.*, 1996). However it's crystal structure has not been reported yet.

In the title compound (Scheme 1, Fig.1), the pyrimidyl ring and dimethylamino group lie in the same plane with a dihedral angle of 1.6 (1)°. This coplanarity may be assisted by the conjugation of π-electrons between pyrimidyl group and nitrogen atom of dimethylamino group. The dihedral angle between the mean plane of the pyrimidyl ring(C1/N1/C2/N2/C3/C4) and the mean plane of the carbamate (O1/O2/C9/N4) is 83.42 (5)° (Fig.1). All bond lengths and bond angles are normal and comparable to those observed in similar structures (Dalpozzo *et al.*, 2001; Madre *et al.*, 2008).

In the crystal structure, weak C—H···O hydrogen bonds are observed [C5—H5C···O2; H5C···O2 = 2.60 Å; C5—H5C···O2 = 163°; C5···O2 = 3.549 (2) Å; -*x* + 1, -*y* + 1, -*z* + 1 and C10—H10C···O2; H10C···O2 = 2.51 Å; C10—H10C···O2 = 157°; C10···O2 = 3.431 (2) Å; *x*,-*y* + 3/2,*z* + 1/2] (Fig. 2).

### Experimental

The title compound was purchased from the Dr. Ehrenstorfer GmbH Company. Slow evaporation of a solution in CH_2_Cl_2_ gave single crystals suitable for X-ray analysis.

### Refinement

All H-atoms were positioned geometrically and refined using a riding model with d(C—H) = 0.98 Å, *U*_iso_ = 1.5*U*_eq_(C) for the H atoms of the methyl groups.

### Crystal data

**Table:**

C_11_H_18_N_4_O_2_	*F*(000) = 512
*M**_r_* = 238.29	*D*_x_ = 1.258 Mg m^−^^3^
Monoclinic, *P*2_1_/*c*	Mo *K*α radiation, λ = 0.71073 Å
Hall symbol: -P 2ybc	Cell parameters from 3964 reflections
*a* = 13.5607 (7) Å	θ = 3.1–28.1°
*b* = 7.7868 (4) Å	µ = 0.09 mm^−^^1^
*c* = 13.1323 (7) Å	*T* = 173 K
β = 114.907 (3)°	Block, colorless
*V* = 1257.72 (11) Å^3^	0.29 × 0.25 × 0.11 mm
*Z* = 4	

### Data collection

**Table:**

Bruker APEXII CCD diffractometer	3093 independent reflections
Radiation source: fine-focus sealed tube	2390 reflections with *I* > 2σ(*I*)
graphite	*R*_int_ = 0.031
Detector resolution: 10.0 pixels mm^-1^	θ_max_ = 28.3°, θ_min_ = 1.7°
φ and ω scans	*h* = −17→18
Absorption correction: multi-scan (*SADABS*; Sheldrick, 1996)	*k* = −10→10
*T*_min_ = 0.975, *T*_max_ = 0.990	*l* = −15→17
11979 measured reflections	

### Refinement

**Table:**

Refinement on *F*^2^	Primary atom site location: structure-invariant direct methods
Least-squares matrix: full	Secondary atom site location: difference Fourier map
*R*[*F*^2^ > 2σ(*F*^2^)] = 0.048	Hydrogen site location: inferred from neighbouring sites
*wR*(*F*^2^) = 0.146	H-atom parameters constrained
*S* = 1.05	*w* = 1/[σ^2^(*F*_o_^2^) + (0.0757*P*)^2^ + 0.3375*P*] where *P* = (*F*_o_^2^ + 2*F*_c_^2^)/3
3093 reflections	(Δ/σ)_max_ = 0.001
160 parameters	Δρ_max_ = 0.27 e Å^−^^3^
0 restraints	Δρ_min_ = −0.26 e Å^−^^3^

### Special details

**Table:**

Geometry. All e.s.d.'s (except the e.s.d. in the dihedral angle between two l.s. planes) are estimated using the full covariance matrix. The cell e.s.d.'s are taken into account individually in the estimation of e.s.d.'s in distances, angles and torsion angles; correlations between e.s.d.'s in cell parameters are only used when they are defined by crystal symmetry. An approximate (isotropic) treatment of cell e.s.d.'s is used for estimating e.s.d.'s involving l.s. planes.
Refinement. Refinement of *F*^2^ against ALL reflections. The weighted *R*-factor *wR* and goodness of fit *S* are based on *F*^2^, conventional *R*-factors *R* are based on *F*, with *F* set to zero for negative *F*^2^. The threshold expression of *F*^2^ > σ(*F*^2^) is used only for calculating *R*-factors(gt) *etc*. and is not relevant to the choice of reflections for refinement. *R*-factors based on *F*^2^ are statistically about twice as large as those based on *F*, and *R*- factors based on ALL data will be even larger.

### Fractional atomic coordinates and isotropic or equivalent isotropic displacement parameters (Å^2^)

**Table:**

	*x*	*y*	*z*	*U*_iso_*/*U*_eq_	
O1	0.24024 (9)	0.53521 (13)	0.53519 (9)	0.0354 (3)	
O2	0.32335 (9)	0.74531 (13)	0.48118 (9)	0.0370 (3)	
N1	0.14628 (10)	0.43992 (15)	0.35622 (10)	0.0312 (3)	
N2	0.22281 (10)	0.23436 (15)	0.27478 (10)	0.0294 (3)	
N3	0.05012 (11)	0.34353 (18)	0.17468 (11)	0.0402 (3)	
N4	0.27470 (12)	0.78741 (17)	0.62525 (11)	0.0385 (3)	
C1	0.23902 (12)	0.43542 (17)	0.44613 (12)	0.0293 (3)	
C2	0.14204 (11)	0.33785 (18)	0.27129 (12)	0.0296 (3)	
C3	0.31494 (11)	0.23573 (17)	0.36835 (12)	0.0287 (3)	
C4	0.32953 (12)	0.33878 (18)	0.46103 (12)	0.0302 (3)	
C5	0.40316 (13)	0.1190 (2)	0.36877 (15)	0.0417 (4)	
H5A	0.3872	0.0846	0.2916	0.063*	
H5B	0.4068	0.0166	0.4137	0.063*	
H5C	0.4730	0.1794	0.4013	0.063*	
C6	0.43355 (14)	0.3450 (2)	0.56746 (13)	0.0416 (4)	
H6A	0.4265	0.2707	0.6243	0.062*	
H6B	0.4474	0.4632	0.5955	0.062*	
H6C	0.4942	0.3049	0.5516	0.062*	
C7	0.03605 (14)	0.2330 (2)	0.08023 (14)	0.0444 (4)	
H7A	−0.0065	0.1316	0.0811	0.067*	
H7B	0.1074	0.1967	0.0861	0.067*	
H7C	−0.0023	0.2961	0.0099	0.067*	
C8	−0.04504 (14)	0.4381 (3)	0.16659 (16)	0.0493 (4)	
H8A	−0.0234	0.5248	0.2262	0.074*	
H8B	−0.0972	0.3587	0.1749	0.074*	
H8C	−0.0788	0.4948	0.0932	0.074*	
C9	0.28291 (11)	0.69672 (18)	0.54274 (11)	0.0292 (3)	
C10	0.22081 (15)	0.7283 (2)	0.69413 (14)	0.0451 (4)	
H10A	0.2093	0.6039	0.6851	0.068*	
H10B	0.1505	0.7863	0.6705	0.068*	
H10C	0.2664	0.7548	0.7731	0.068*	
C11	0.31354 (17)	0.9642 (2)	0.64121 (17)	0.0536 (5)	
H11A	0.3535	0.9857	0.5953	0.080*	
H11B	0.3618	0.9830	0.7205	0.080*	
H11C	0.2514	1.0427	0.6185	0.080*	

### Atomic displacement parameters (Å^2^)

**Table:**

	*U*^11^	*U*^22^	*U*^33^	*U*^12^	*U*^13^	*U*^23^
O1	0.0517 (7)	0.0303 (5)	0.0323 (6)	−0.0064 (4)	0.0258 (5)	−0.0063 (4)
O2	0.0458 (6)	0.0318 (5)	0.0394 (6)	−0.0063 (4)	0.0236 (5)	−0.0044 (4)
N1	0.0350 (6)	0.0273 (6)	0.0343 (7)	−0.0024 (5)	0.0174 (5)	−0.0023 (5)
N2	0.0350 (6)	0.0252 (6)	0.0289 (6)	−0.0022 (5)	0.0145 (5)	−0.0024 (5)
N3	0.0341 (7)	0.0422 (7)	0.0370 (7)	−0.0009 (6)	0.0078 (6)	−0.0073 (6)
N4	0.0487 (8)	0.0343 (7)	0.0350 (7)	0.0003 (6)	0.0200 (6)	−0.0089 (5)
C1	0.0415 (8)	0.0230 (6)	0.0286 (7)	−0.0063 (5)	0.0199 (6)	−0.0022 (5)
C2	0.0327 (7)	0.0258 (6)	0.0315 (7)	−0.0052 (5)	0.0148 (6)	−0.0007 (5)
C3	0.0348 (7)	0.0228 (6)	0.0301 (7)	−0.0024 (5)	0.0151 (6)	0.0010 (5)
C4	0.0371 (7)	0.0259 (7)	0.0275 (7)	−0.0026 (5)	0.0135 (6)	−0.0004 (5)
C5	0.0403 (8)	0.0411 (9)	0.0411 (9)	0.0074 (7)	0.0146 (7)	−0.0054 (7)
C6	0.0461 (9)	0.0405 (8)	0.0305 (8)	0.0011 (7)	0.0088 (7)	−0.0018 (7)
C7	0.0426 (9)	0.0520 (10)	0.0332 (8)	−0.0093 (8)	0.0107 (7)	−0.0084 (7)
C8	0.0358 (9)	0.0564 (11)	0.0496 (10)	0.0051 (7)	0.0120 (8)	0.0006 (8)
C9	0.0311 (7)	0.0275 (7)	0.0259 (7)	0.0024 (5)	0.0090 (6)	−0.0015 (5)
C10	0.0606 (11)	0.0501 (10)	0.0299 (8)	0.0136 (8)	0.0242 (8)	−0.0006 (7)
C11	0.0651 (12)	0.0372 (9)	0.0581 (11)	−0.0025 (8)	0.0257 (10)	−0.0191 (8)

### Geometric parameters (Å, °)

**Table:**

O1—C9	1.3704 (18)	C5—H5B	0.9800
O1—C1	1.3985 (16)	C5—H5C	0.9800
O2—C9	1.2128 (18)	C6—H6A	0.9800
N1—C1	1.3138 (19)	C6—H6B	0.9800
N1—C2	1.3510 (18)	C6—H6C	0.9800
N2—C3	1.3333 (19)	C7—H7A	0.9800
N2—C2	1.3449 (19)	C7—H7B	0.9800
N3—C2	1.3540 (19)	C7—H7C	0.9800
N3—C8	1.450 (2)	C8—H8A	0.9800
N3—C7	1.455 (2)	C8—H8B	0.9800
N4—C9	1.3367 (19)	C8—H8C	0.9800
N4—C10	1.456 (2)	C10—H10A	0.9800
N4—C11	1.457 (2)	C10—H10B	0.9800
C1—C4	1.381 (2)	C10—H10C	0.9800
C3—C4	1.401 (2)	C11—H11A	0.9800
C3—C5	1.501 (2)	C11—H11B	0.9800
C4—C6	1.512 (2)	C11—H11C	0.9800
C5—H5A	0.9800		
			
C9—O1—C1	115.24 (11)	C4—C6—H6C	109.5
C1—N1—C2	114.71 (12)	H6A—C6—H6C	109.5
C3—N2—C2	117.40 (12)	H6B—C6—H6C	109.5
C2—N3—C8	121.66 (14)	N3—C7—H7A	109.5
C2—N3—C7	121.19 (13)	N3—C7—H7B	109.5
C8—N3—C7	116.42 (13)	H7A—C7—H7B	109.5
C9—N4—C10	124.84 (14)	N3—C7—H7C	109.5
C9—N4—C11	117.86 (14)	H7A—C7—H7C	109.5
C10—N4—C11	117.05 (14)	H7B—C7—H7C	109.5
N1—C1—C4	126.79 (13)	N3—C8—H8A	109.5
N1—C1—O1	113.98 (13)	N3—C8—H8B	109.5
C4—C1—O1	119.16 (13)	H8A—C8—H8B	109.5
N2—C2—N1	124.99 (13)	N3—C8—H8C	109.5
N2—C2—N3	117.82 (13)	H8A—C8—H8C	109.5
N1—C2—N3	117.17 (13)	H8B—C8—H8C	109.5
N2—C3—C4	122.69 (13)	O2—C9—N4	126.22 (14)
N2—C3—C5	115.77 (13)	O2—C9—O1	122.26 (12)
C4—C3—C5	121.55 (13)	N4—C9—O1	111.52 (13)
C1—C4—C3	113.39 (13)	N4—C10—H10A	109.5
C1—C4—C6	122.76 (13)	N4—C10—H10B	109.5
C3—C4—C6	123.85 (14)	H10A—C10—H10B	109.5
C3—C5—H5A	109.5	N4—C10—H10C	109.5
C3—C5—H5B	109.5	H10A—C10—H10C	109.5
H5A—C5—H5B	109.5	H10B—C10—H10C	109.5
C3—C5—H5C	109.5	N4—C11—H11A	109.5
H5A—C5—H5C	109.5	N4—C11—H11B	109.5
H5B—C5—H5C	109.5	H11A—C11—H11B	109.5
C4—C6—H6A	109.5	N4—C11—H11C	109.5
C4—C6—H6B	109.5	H11A—C11—H11C	109.5
H6A—C6—H6B	109.5	H11B—C11—H11C	109.5
			
C2—N1—C1—C4	0.0 (2)	N1—C1—C4—C3	−1.1 (2)
C2—N1—C1—O1	−177.02 (11)	O1—C1—C4—C3	175.74 (11)
C9—O1—C1—N1	−95.73 (15)	N1—C1—C4—C6	178.91 (14)
C9—O1—C1—C4	87.02 (16)	O1—C1—C4—C6	−4.2 (2)
C3—N2—C2—N1	−1.6 (2)	N2—C3—C4—C1	1.0 (2)
C3—N2—C2—N3	176.89 (13)	C5—C3—C4—C1	−178.95 (13)
C1—N1—C2—N2	1.5 (2)	N2—C3—C4—C6	−179.06 (14)
C1—N1—C2—N3	−177.02 (13)	C5—C3—C4—C6	1.0 (2)
C8—N3—C2—N2	173.88 (15)	C10—N4—C9—O2	176.80 (15)
C7—N3—C2—N2	4.0 (2)	C11—N4—C9—O2	2.7 (2)
C8—N3—C2—N1	−7.5 (2)	C10—N4—C9—O1	−3.9 (2)
C7—N3—C2—N1	−177.33 (13)	C11—N4—C9—O1	−178.05 (14)
C2—N2—C3—C4	0.3 (2)	C1—O1—C9—O2	−4.7 (2)
C2—N2—C3—C5	−179.80 (13)	C1—O1—C9—N4	175.97 (12)

### Hydrogen-bond geometry (Å, °)

**Table:**

*D*—H···*A*	*D*—H	H···*A*	*D*···*A*	*D*—H···*A*
C5—H5C···O2^i^	0.98	2.60	3.549 (2)	163
C10—H10C···O2^ii^	0.98	2.51	3.431 (2)	157

Symmetry codes: (i) −*x*+1, −*y*+1, −*z*+1; (ii) *x*, −*y*+3/2, *z*+1/2.
